# Study of GaN-Based Thermal Decomposition in Hydrogen Atmospheres for Substrate-Reclamation Processing

**DOI:** 10.3390/ma11112082

**Published:** 2018-10-24

**Authors:** Shih-Yung Huang, Jian-Cheng Lin, Sin-Liang Ou

**Affiliations:** 1 Department of Industrial Engineering and Management, Da-Yeh University, Changhua 515, Taiwan; 2 Bachelor Program for Design and Materials for Medical Equipment and Device, Da-Yeh University, Changhua 515, Taiwan; qwas7182004@gmail.com (J.-C.L.); slo@mail.dyu.edu.tw (S.-L.O.)

**Keywords:** GaN, GaO_2_H, nanostructures, thermal decomposition, substrate-reclamation

## Abstract

This study investigates the thermal decomposition behavior of GaN-based epilayers on patterned sapphire substrates (GaN-epi/PSSs) in a quartz furnace tube under a hydrogen atmosphere. The GaN-epi/PSS was decomposed under different hydrogen flow rates at 1200 °C, confirming that the hydrogen flow rate influences the decomposition reaction of the GaN-based epilayer. The GaN was completely removed and the thermal decomposition process yielded gallium oxyhydroxide (GaO_2_H) nanostructures. When observed by transmission electron microscopy (TEM), the GaO_2_H nanostructures appeared as aggregates of many nanograins sized 2–5 nm. The orientation relationship, microstructure, and formation mechanism of the GaO_2_H nanostructures were also investigated.

## 1. Introduction

GaN and its alloys have been widely used in optoelectronic devices such as green- to deep-ultraviolet light-emitting diodes [1,2,3] and photodetectors [4,5,6]. Various GaN-based semiconductors have become commercially available, although the large number of wafers that are scrapped during the manufacturing process remains problematic. It is hoped that reusing the sapphire substrate of epitaxial layers reclaimed from scrapped wafers will reduce the manufacturing costs. For substrate reclamation, thermal decomposition is more suitable than the common chemical mechanical polishing method, because the former is simple and cheap. However, the thermal decomposition behavior of GaN has been little investigated. In 1965, Munir and Searcy [7] investigated the heat of activation of the reaction 2GaN_(s)_ = 2Ga_(l)_ + N_2(g)_. The decomposition of GaN in the presence of H_2_ was first studied by Thurmond and Logan in the 1970s [8,9]. Later, GaN decomposition in the presence of H_2_, N_2_, HCl, and NH_3_ at different temperatures and pressures was reported [10,11,12,13,14,15,16]. However, in these reports, the decomposition of GaN epilayers was either prohibited, suppressed, or delayed, and the complete GaN decomposition was not extensively studied.

In this study, we investigate the behaviors of GaN-based thermal decomposition under hydrogen atmospheres with different flow rates. We quantify the partial and complete decompositions of the GaN-based layers, and compare the surface morphologies and crystalline phases of the decomposition products. We also explore the mechanism of the GaN-based thermal decomposition under a hydrogen atmosphere.

## 2. Experimental Procedures

**Figure 1:**
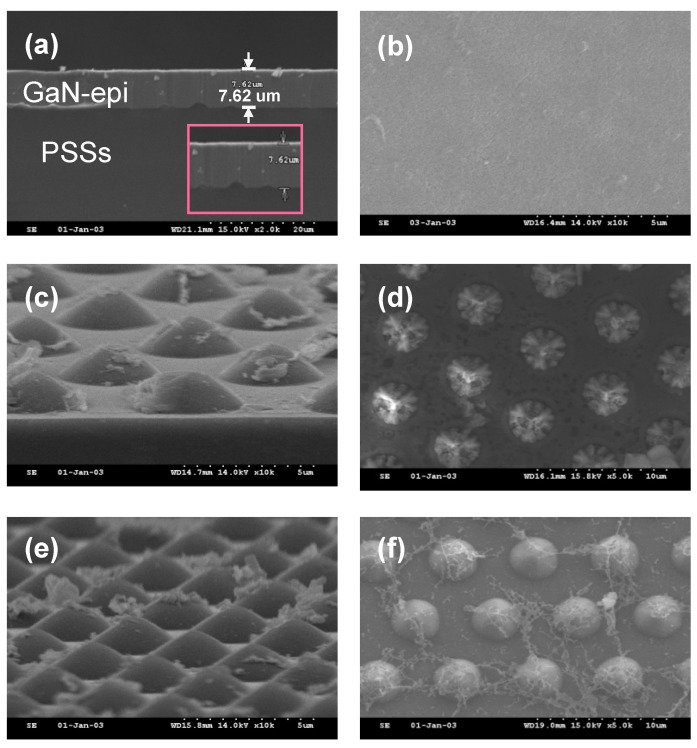
Cross-sectional and plane-view SEM images of the GaN-based epilayers on patterned sapphire substrates (GaN-epi/PSSs) morphologies under H_2_ gas flowing at (**a**,**b**) 0 cm^3^/min, (**c**,**d**) 10 cm^3^/min, and (**e**,**f**) 25 cm^3^/min at 1200 °C for 3 h. The enlargement enclosed by the pink frame in panel (**a**) depicts a clear GaN-epi/PSS structure.

The sample was composed of GaN-based epilayers on patterned sapphire substrates (GaN-epi/PSSs) grown by the standard GaN-epi/PSS fabrication procedure. The original GaN-epi/PSSs was 7.62 μm thick, as shown in Figure 1a. The GaN-epi surfaces were decomposed by loading the sample into a furnace preheated to 1200 °C and filled with hydrogen gas flowing at 0, 10, or 25 cm^3^/min. The decomposition time was 3 h.

The surface morphologies and compositions of the decomposed GaN-epi/PSS sample were observed with a scanning electron microscopy–energy dispersive spectrometer (SEM–EDS, S-3000H, Hitachi, Tokyo, Japan). The crystal structures of the GaN-epi/PSSs were characterized by X-ray diffraction (XRD; PANalytical, X’Pert Pro MRD System, Malvern Panalytical, Malvern, UK). The XRD radiation source was the Cu Kα line (*λ* = 1.541874), and the monochromator was Ge (220). The orientation relationships and microstructures of the GaN-epi/PSSs samples were investigated under a transmission electron microscope (TEM) (JEOL JEM-2100F, Tokyo, Japan).

## 3. Results and Discussion

Figure 1 shows SEM images of the GaN-epi/PSSs morphologies after 3 h in the 1200 °C furnace with different gas-flow rates. Under the gas-flow rate of 10 cm^3^/min, the thermal decomposition yielded PSS shapes (Figure 1c,d). When the gas-flow rate was increased to 25 cm^3^/min, the surface shape better resembled an exposed PSS than at 10 cm^3^/min (Figure 1e,f). These results prove that the decomposition reaction of the GaN-epi/PSSs depended on the hydrogen flow rate. Previously, the GaN decomposition process was found to strongly depend on the temperature and ambient conditions [17]. It is worth noting that the present GaN films were completely decomposed, forming GaO_2_H on the exposed PSS surface (Figure 1e,f). The GaO_2_H reactant was confirmed by SEM–EDS and XRD, as discussed below. Later, this phenomenon will be further investigated by the TEM technique.

**Figure 2:**
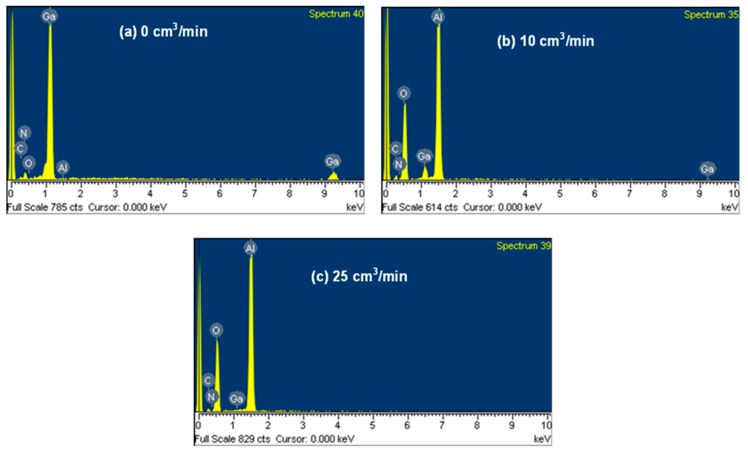
Scanning electron microscopy–energy dispersive spectrometer (SEM–EDS) spectra of the GaN-epi/PSSs decomposed at 1200 °C for 3 h under hydrogen flow rates of (**a**) 0 cm^3^/min, (**b**) 10 cm^3^/min, and (**c**) 25 cm^3^/min.

**Figure 3:**
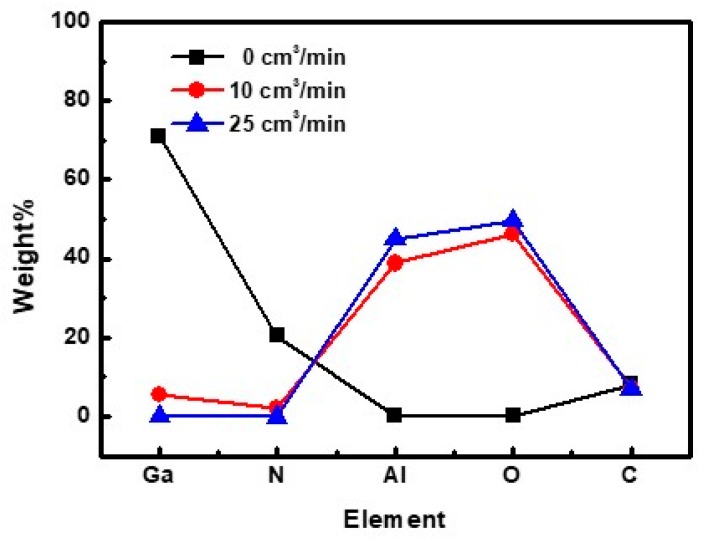
Quantitative SEM–EDS results of the GaN-epi/PSSs decomposed at 1200 °C for 3 h under hydrogen flow rates of 0, 10, and 25 cm^3^/min.

Figure 2 shows the SEM–EDS spectra of the GaN-epi/PSSs decomposed in the 1200 °C furnace under hydrogen flow rates of 0, 10, and 25 cm^3^/min. In the absence of H_2_ gas (0 cm^3^/min), the GaN-epi/PSSs was overwhelmingly dominated by Ga and N elements (Figure 2a). Increasing the H_2_ flow rate gradually decreased the amounts of Ga and N elements, and showed the existence of the Al and O elements (Figure 2b,c). Under the high flow rate (25 cm^3^/min), the PSS was completely recovered by the decomposition process, and the Ga and N compositions had vanished (Figure 2c). The carbon peaks in the spectra are attributable to atmospheric carbon adsorbed to the sample surface. The weight percentages (wt.%) of the SEM–EDS for the quantitative analysis are compared in Figure 3 and Table 1. The Al and O amounts increased with increasing gas flow rate. The Ga content (0.12%) after decomposition under 25 cm^3^/min H_2_ gas was attributed to the solid GaO_2_H reactant on the exposed PSS surface.

**Figure 4:**
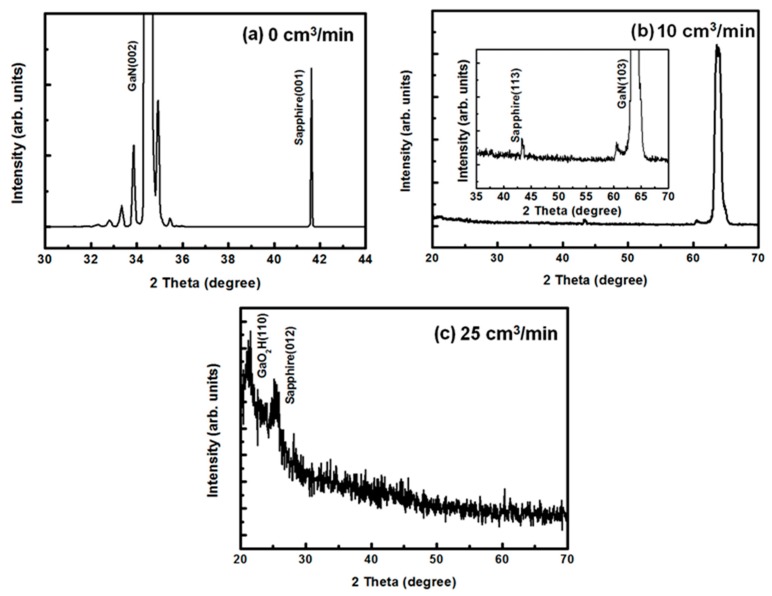
XRD spectra of the GaN-epi/PSSs decomposed at 1200 °C for 3 h under gas-flow rates of (**a**) 0 cm^3^/min, (**b**) 10 cm^3^/min, and (**c**) 25 cm^3^/min.

Figure 4 shows the XRD spectra of the GaN-epi/PSSs decomposed under gas flow rates of 0, 10, and 25 cm^3^/min. In the absence of H_2_ gas (0 cm^3^/min; Figure 4a), the XRD spectrum shows a GaN (002) peak located at approximately 2θ = 34.6° and several satellite peaks from InGaN/GaN multiple quantum well structures. The sapphire (001) phase near 41.6° also appears. Under the lower gas flow (10 cm^3^/min; Figure 4b), sapphire (113) and GaN (103) peaks are observed at approximately 43.4° and 63.6°, respectively. The GaN (103) peak is strong, but the GaN (002) peak and several satellite peaks have disappeared, indicating that the GaN film had gradually decomposed. Increasing the gas flow rate to 25 cm^3^/min completely removed the GaN phase, as shown in Figure 4c. The result implies that the GaN-epi/PSSs was completely decomposed under sufficiently high gas flow rates. These phenomena well agree with the results in Figure 2. Notably, in the grazing angle-XRD spectra under 25 cm^3^/min gas flow, the GaO_2_H (110) peak was located at 21.47°, indicating that GaO_2_H was present on the exposed PSS surface after the GaN had decomposed. In our previous study, the chemical reaction of the GaN decomposition was described as [18]

GaN_(s)_ + H_2(v)_ + 1/2 O_2(v)_ + 1/4 N_2(v)_ = 1/2 Ga_(v)_ + 1/2 GaO_2_H_(s)_ + 1/2 N_2(v)_ + 1/2 NH_3(v)_ (1)

**Figure 5:**
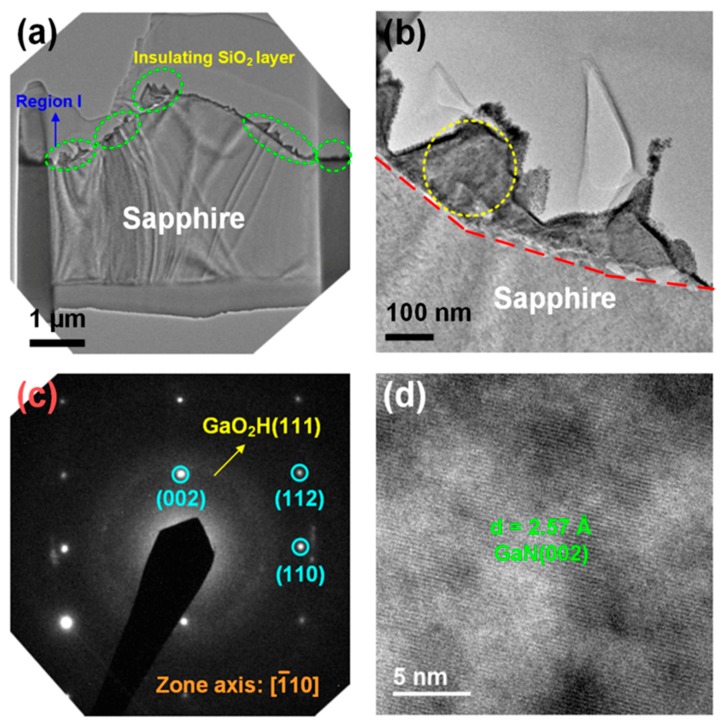
(**a**) Cross-sectional transmission electron microscopy (TEM) image of the GaN-epi/PSS sample decomposed at 1200 °C for 3 h under a gas-flow rate of 10 cm^3^/min; (**b**) TEM image of region I taken at higher magnification; and (**c**,**d**) SAED pattern of region I and an high resolution transmission electron microscopy (HR–TEM) image, respectively, focused on the yellow-circled area in (**b**).

The microstructures of the GaN-epi/PSSs after decomposition were investigated by TEM. The samples prepared for decomposition at 1200 °C for 3 h under gas flows of 10 and 25 cm^3^/min were analysed sequentially. Figure 5a shows the cross-sectional TEM image of the GaN-epi/PSS sample decomposed under 10 cm^3^/min. Several residues (marked with green circles) appear on the bottom, sidewall, and top areas of the pattern. One of these residues (marked as region I) was selected for further observation. Moreover, the insulating SiO_2_ layer on these residues was deposited for the focused ion beam (FIB) process. Figure 5b is a higher-resolution TEM image of region I, which clarifies the interface between the region and the sapphire. Note that the residues were irregularly shaped. The selected area electron diffraction (SAED) pattern of region I (focused on the area delineated by the yellow circle in Figure 5b) is presented in Figure 5c. The regularly arranged diffraction dots reveal a single crystal structure of the residues. After analysis of this single-crystalline diffraction pattern, the residue was identified as GaN epilayer grown along the [002] direction (with the $\left[\bar{1}10\right]$ zone axis). A ring diffraction pattern with very weak intensity also appeared (Figure 5c). The ring pattern was indexed to the GaO_2_H (111) phase, suggesting that GaO_2_H nanocrystals were formed in the residue. Figure 5d is a high resolution TEM (HR-TEM) image of the area enclosed by the yellow circle in Figure 5b. The image shows a regular lattice arrangement. The *d*-spacing was determined as 2.57 Å and was identified as the GaN (002) plane. Obviously, even at the lower gas flow rate (10 cm^3^/min), GaN residues existed on the PSS. Based on the above-phenomenon, the GaN was only partially decomposed under gas flows of 10 cm^3^/min, and the small amount of GaO_2_H was formed on the GaN surface of the incompletely decomposed. Consequently, none of the GaO_2_H phases were detected by XRD (see Figure 4b).

**Figure 6:**
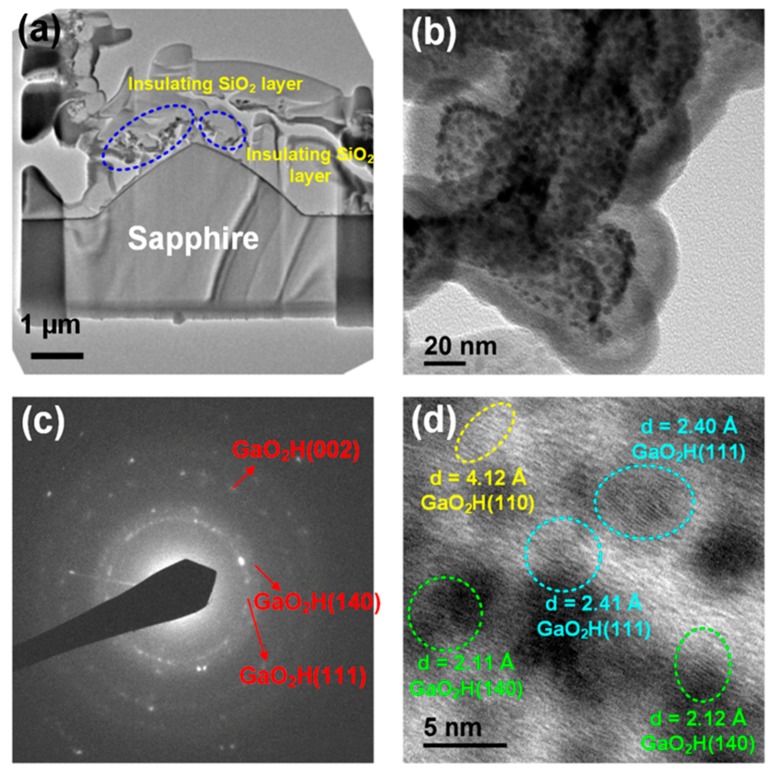
(**a**) Cross-sectional TEM image of the GaN-epi/PSS sample decomposed at 1200 °C for 3 h under a gas-flow rate of 25 cm^3^/min; (**b**) TEM image of the nanostructures taken at higher magnification; and (**c**,**d**) SAED pattern and an HR–TEM image of the nanostructures, respectively.

Figure 6 displays the TEM results of the GaN-epi/PSSs sample decomposed at 1200 °C under the high gas flow rate (25 cm^3^/min). As shown in the cross-sectional TEM image (Figure 6a), there was less residue on this sample than on the sample decomposed under the 10 cm^3^/min flow (Figure 5a). The insulating SiO_2_ layer also can be found in Figure 6a. Moreover, several nanostructures were formed during the decomposition process (marked with blue circles in the image). The morphology of the nanostructures can be observed in the plane-view SEM image (Figure 1f). The same nanostructures were found in the sample decomposed under the 10 cm^3^/min flow. During the FIB process, the nanostructures were easily broken and disappeared from the surface. Thus, almost no nanostructure is visible in Figure 5. To understand the formation of these nanostructures, we observed them under a higher TEM magnification (Figure 6b). Interestingly, these nanostructures were aggregates of many nanograins of size 2–5 nm. Figure 6c shows the SAED pattern of the nanostructures. The three diffraction rings were indexed to GaO_2_H (111), GaO_2_H (140), and GaO_2_H (002) phases. All rings were of very low brightness, indicating the poor crystal quality of the phases. Consequently, none of these phases were detected by XRD (see Figure 4). Although the GaO_2_H (110) diffraction peak appeared in the XRD pattern of Figure 4, no GaO_2_H (110) electron diffraction ring is visible in Figure 6c. We expected that the GaO_2_H (110) ring was close to the centre of the electron diffraction pattern, and was obscured by the very bright electron beam. Figure 6d is an HR-TEM image of the nanostructures. Several nano grains with various *d*-spacings were identified as GaO_2_H (110), GaO_2_H (111), and GaO_2_H (140) phases, again confirming that GaO_2_H (110) phase formed in the decomposed sample. A substrate can be completely reclaimed by removing the GaO_2_H reactant in alkaline solution [18].

**Figure 7:**
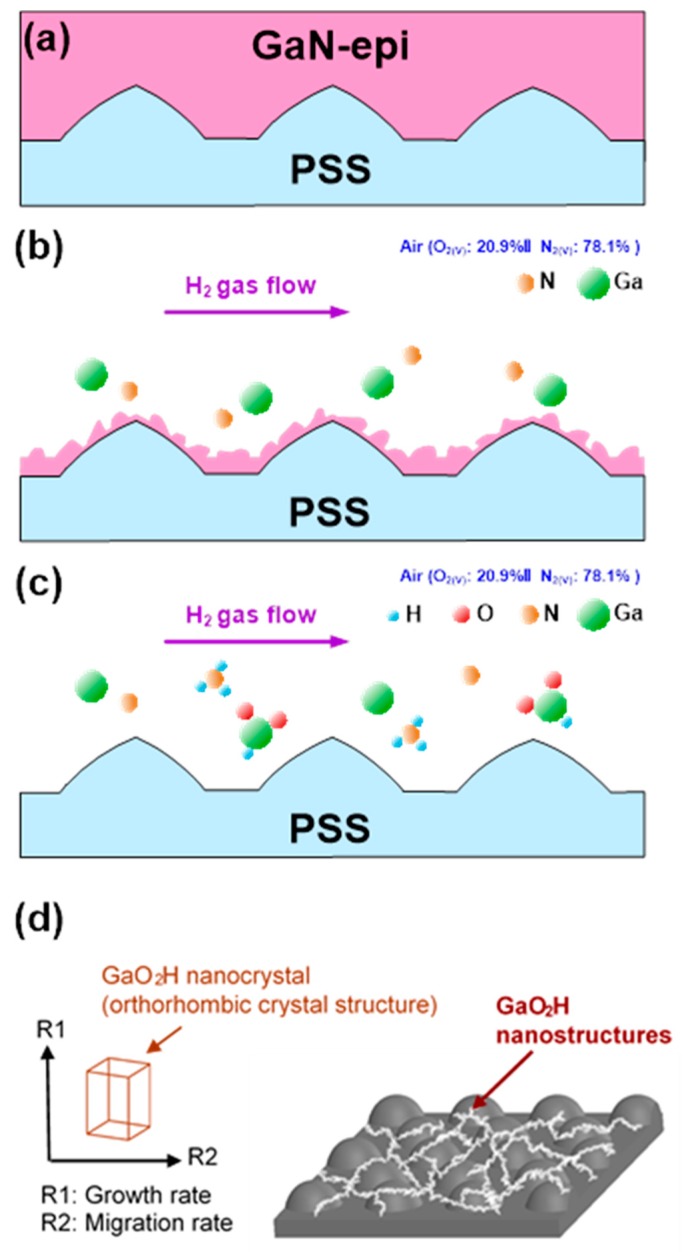
Schematic diagram for (**a**) a GaN-epi/PSS structure, (**b**) the H_2_ chemically reacted with GaN, releasing nitrogen atoms and leaving the Ga atoms, (**c**) Some of the Ga atoms reacted chemically with the O atoms in the air and the loading gas H_2_, forming GaO_2_H nanocrystals. Meanwhile, some of the N atoms reacted with H_2_ to produce NH_3_, and (**d**) the remaining GaO_2_H nanocrystals were formed a discontinuous and uneven GaO_2_H nanostructure on the exposed PSS surface.

The behavior of GaN-based thermal decomposition in the hydrogen atmosphere is shown in Figure 7. When the GaN-epi/PSS sample was treated with hydrogen at high temperature (1200 °C), the hydrogen molecules began diffusing and adsorbing onto the GaN surface. Once adsorbed, they chemically reacted with GaN, releasing nitrogen atoms from the surface and leaving the Ga atoms. This mechanism is schematized in Figure 7a,b. Some of the Ga atoms reacted chemically with the O atoms in the air and the loading gas H_2_, forming GaO_2_H nanocrystals. Meanwhile, some of the N atoms reacted with H_2_ to produce NH_3_. The NH_3_ byproduct and the unreacted gas molecules (Ga, N_2_, and the loading gas H_2_) were expelled from the reaction chamber by forced convection, leaving only the GaO_2_H nanocrystals (Figure 7c). The remaining GaO_2_H nanocrystals were formed a discontinuous and uneven GaO_2_H nanostructure on the exposed PSS surface (see Figure 7d). This behavior was attributed to the smaller grains sized, the lower surface growth rate than migration rate of the GaO_2_H nanocrystals, and the surface migration distance lengthens at the high temperature. It is worth mentioning that the crystal structure of the InGaN well layers was a small amount of In doped to GaN and only partially replaced the crystal position of Ga. Therefore, the behavior of the InGaN thermal decomposition in the H_2_ atmosphere was similar to that of the GaN thermal decomposition. The H_2_ chemically reacted with InGaN, releasing nitrogen atoms and leaving the In and Ga atoms, and the unreacted In and Ga gas molecules were expelled from the reaction chamber.

## 4. Conclusions

We completely decomposed GaN under a hydrogen atmosphere, and discussed the decomposition mechanism. Hydrogen promoted the decomposition of GaN into Ga and N atoms; the Ga atoms then reacted with oxygen in the atmosphere and the hydrogen loading gas to form GaO_2_H nanocrystals. Interestingly, these GaO_2_H nanocrystals formed a discontinuous and uneven GaO_2_H nanostructure on the exposed PSS surface. This behavior was attributed to the smaller grains size, the lower surface-growth rate than migration rate of the GaO_2_H nanocrystals, and the surface migration distance lengthens at the high temperature. A complete substrate reclamation process is possible by removing the GaO_2_H reactant using alkaline solution. The decomposition mechanism is potentially applicable to the reclamation of sapphire substrates in the GaN-based semiconductor industry.

## Figures and Tables

**Table 1:** Compositions of the GaN-epi/PSSs under different gas flow rates, obtained by EDS.

Gas-Flow Rates (cm^3^/min)	0	10	25
	(wt.%)	(wt.%)	(wt.%)
Ga atom	71.13	5.56	0.12
N atom	20.43	2.14	0
Al atom	0	38.81	45
O atom	0	46.33	49.67
C atom	8.18	7.16	6.8
